# Unraveling the Multifaceted Nature of CD8 T Cell Exhaustion Provides the Molecular Basis for Therapeutic T Cell Reconstitution in Chronic Hepatitis B and C

**DOI:** 10.3390/cells10102563

**Published:** 2021-09-28

**Authors:** Valeria Barili, Andrea Vecchi, Marzia Rossi, Ilaria Montali, Camilla Tiezzi, Amalia Penna, Diletta Laccabue, Gabriele Missale, Paola Fisicaro, Carolina Boni

**Affiliations:** 1 Laboratory of Viral Immunopathology, Unit of Infectious Diseases and Hepatology, Azienda Ospedaliero-Universitaria di Parma, 43126 Parma, Italy; barili.valeria@gmail.com (V.B.); avecchi2@ao.pr.it (A.V.); rossi.marzia@gmail.com (M.R.); ilaria.montali@unipr.it (I.M.); camilla.tiezzi@studenti.unipr.it (C.T.); apenna@ao.pr.it (A.P.); diletta.laccabue@unipr.it (D.L.); gabriele.missale@unipr.it (G.M.); 2 Department of Medicine and Surgery, University of Parma, 43126 Parma, Italy

**Keywords:** chronic hepatitis, T cell exhaustion, immune-modulation, HBV, HCV

## Abstract

In chronic hepatitis B and C virus infections persistently elevated antigen levels drive CD8+ T cells toward a peculiar differentiation state known as T cell exhaustion, which poses crucial constraints to antiviral immunity. Available evidence indicates that T cell exhaustion is associated with a series of metabolic and signaling deregulations and with a very peculiar epigenetic status which all together lead to reduced effector functions. A clear mechanistic network explaining how intracellular metabolic derangements, transcriptional and signaling alterations so far described are interconnected in a comprehensive and unified view of the T cell exhaustion differentiation profile is still lacking. Addressing this issue is of key importance for the development of innovative strategies to boost host immunity in order to achieve viral clearance. This review will discuss the current knowledge in HBV and HCV infections, addressing how innate immunity, metabolic derangements, extensive stress responses and altered epigenetic programs may be targeted to restore functionality and responsiveness of virus-specific CD8 T cells in the context of chronic virus infections.

## 1. Introduction

Therapy for chronic hepatitis B (CHB) relies on direct acting antiviral drugs, that suppress virus production but do not eradicate HBV from the liver, requiring lifelong treatments [1]. Therefore, there is a pressing need to find new therapeutic strategies achieving a definitive cure for CHB. Conversely, direct acting antiviral drugs (DAA) currently available for chronic hepatitis C lead to a sustained virologic response in 85–99% of cases, although a large population of treatment failures still exists [2]. For these reasons, the present review is aimed at addressing the emerging concepts of immune modulation for both chronic hepatitis B and C deriving from the most recent advances in viral pathogenesis.

**Figure 1:**
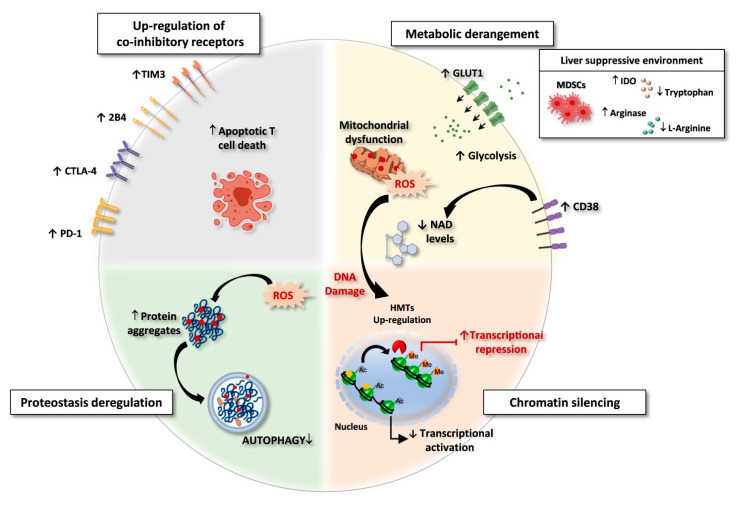
Exhausted CD8 T cells in HBV and HCV chronic infections. Features of exhausted T cells: (i) Upregulation of multiple co-inhibitory receptors; (ii) Metabolic derangement with depolarized mitochondria and ROS accumulation. A suppressive intrahepatic microenvironment enriched with amino acids degrading enzymes (e.g., arginase and IDO/TDO) causes reduction of amino acid availability. Lower respiratory capacity and amino acid starvation favor glycolysis as a compensatory mechanism, with upregulation of the glucose transporter GLUT-1. In addition, over-expression of CD38 can lead to increased intracellular NAD consumption, exacerbating mitochondrial dysfunction. (iii) Chromatin silencing due to histone methyltransferases (HMTs) upregulation and to the reduced availability of acetyl-CoA, as a consequence of low NAD levels, fundamental co-factor for histone acetylation. (iv) Proteostasis deregulation. High ROS levels lead to oxidation and damage of proteins and cell organelles. Exhausted CD8 T cells express a deregulated proteostasis with lower autophagy rates, leading to a defective clearance of aggregated proteins and damaged organelles. Created with BioRender.com, access on 1 September 2021.

In chronic hepatitis B and C, virus-specific T-lymphocytes have been described to be deeply dysfunctional, with severity of their hyporesponsive state at least in part depending upon viremia and antigenemia levels [3] (Figure 1).

Such a dysfunctional state, called “T cell exhaustion”, is characterized by the progressive loss of T cell effector functions, due to the repeated triggering of CD8 T cells caused by persistent exposure to high antigen concentrations [4]. First observed in chronic LCMV infection in mice, it has subsequently been described in different models of animal and human chronic virus infections and in cancer [4]. Different degrees of functional T cell impairment have been identified, with IL-2 production, cytolytic activity, cytokine polyfunctionality and proliferative capacity lost first, followed by TNF-α and IFN-γ secretion, up to physical T cell deletion at the most terminal stages of exhaustion [5]. Besides persistent antigenic stimulation, further extrinsic factors, such as increased regulatory T cell activity, inhibitory cytokines, and the loss of CD4 T cell help have been shown to promote T cell dysfunction [6,7,8,9].

Comparisons of gene expression profiles of virus-specific CD8 T cells derived from acutely or chronically LCMV-infected mice led to the discovery of a high expression of the co-inhibitory receptor PD-1 in chronic infection, and to its role in promoting a dysfunctional phenotype [10]. Later, the co-expression of other up-regulated inhibitory checkpoints has been associated with the exhausted CD8 T cell phenotype [6,11,12,13]. Typically, the higher is the number of inhibitory receptors co-expressed by exhausted T cells, the more severe is the exhaustion state [11]. Additional features of exhausted T cells are an altered transcriptional program, a deregulated metabolism, a loss of memory markers, an up-regulation of some transcription factors, such as Eomes and Blimp-1 [14], with severe T cell exhaustion associated to high Eomes expression [15]. However, it has recently been demonstrated that not only Eomes expression, but also its subcellular localization influences the degree of T cell exhaustion: when present within the nucleus, Eomes regulates PD-1 expression by competing with the transcription factor Tbet for binding to Tbox sequences. Thus, a high Eomes:Tbet ratio can enhance PD-1 expression, since Eomes is a weaker PD-1 repressor than Tbet [16].

Starting from early observations showing that terminally exhausted PD-1^hi^ and less differentiated PD-1^int^ coexist within exhausted LCMV-specific CD8 T cells, it has progressively become clear that exhausted virus-specific CD8 T cells cannot be considered a homogeneously dysfunctional population [17]. Indeed, T cell subsets with different degrees of functionality and different sensitivity to immune modulatory interventions have been detected in different models of chronic viral infections. In addition, recent single cell (sc) RNA-seq data support the concept of a tissue-associated heterogeneity, showing that the transcriptomic profile of exhausted virus-specific CD8 T cells can be shaped by the specific microenvironment where they differentiate [18]. A subset of exhausted T cells expressing the TCF1 transcription factor has been identified as endowed with memory-like characteristics and a higher sensitivity to inhibitory checkpoint blockade than their TCF1-negative counterparts, that is composed of more terminally exhausted T cells [19,20]. Also in chronic hepatitis B, subsets of differentially exhausted virus-specific CD8 T cells have been observed [21,22], among which terminally exhausted cells do not represent the majority of the virus-specific population both at peripheral (personal communication) and at intrahepatic/intratumor level in CHB patients with hepatocellular carcinoma [23]. Moreover, various degrees of T cell dysfunction have been identified for HBV-specific CD8 T cells that recognize different HBV antigens [24]. Notably, PD-1 and CD127 expression levels can allow to identify a subset of PD-1^low^/-CD127^high^ CD8 T cells, which are more sensitive to functional restoration, predicting the in vitro response to immune-modulatory treatments (personal communication) and thus allowing to identify those patients who are more likely to benefit from immune-modulation. Co-expression of CD127, PD-1 and TCF1 has been reported to identify also in chronic hepatitis C a subset of virus-specific CD8 T cells with residual proliferative potential [25]. This memory-like T cell subset is maintained after spontaneous or antiviral therapy-induced resolution of chronic infection, showing that recovery after a long-lasting exposure to HCV cannot lead to a complete reacquisition of phenotypic and function features of canonical memory T cells [25]. In line with this observation, virus-specific T cells maintain a “molecular scar” after resolution of chronic HBV and HCV infections which doesn’t persist following spontaneous control of acute hepatitis [26,27,28,29,30].

Although severe exhaustion is likely incompletely reversible even after complete antigen clearance, functional restoration can apparently be achieved with less terminally exhausted T cells, suggesting that immune-modulation may still be a possible strategy to fight chronic HBV and HCV infections.

**Figure 2:**
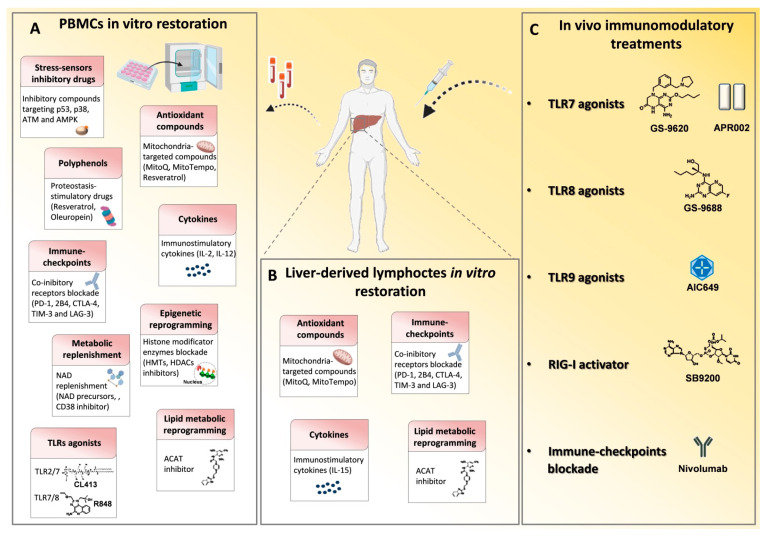
Immune based approaches in viral hepatitis. Many drugs that modulate the immune system have been employed in chronic HBV and HCV infections in order to reconstitute antiviral T cell responses and achieve viral clearance. (**A**) In vitro T cell modulatory treatments tested on patient-derived PBMCs and (**B**) on liver-derived lymphocytes to restore signaling deregulations, cytokine levels, metabolic functions and epigenetic landscape and to block inhibitory receptor signals. (**C**) In vivo T cell function reconstitution interventions are summarized. They target the activation of innate immunity receptors, such as toll like receptors (TLRs) and retinoic acid-inducible gene 1 (RIG-I) by specific agonists and through checkpoint blockade, such as with an anti-PD-1 monoclonal antibody (Nivolumab). p53, tumor-suppressor protein; p38, mitogen-activated protein kinase; ATM, Ataxia Telangiectasia Mutated; AMPK, AMP-activated protein kinase; MitoQ, mitoquinone; MitoTempo, mitochondria-targeted superoxide dismutase; PD-1, programmed cell death 1; 2B4, Natural Killer Cell Receptor 2B4; CTLA4, cytotoxic T lymphocyte antigen 4; TIM3, T cell immunoglobulin mucin receptor 3; LAG3, lymphocyte activation gene 3 protein; HMTs, histone methyltransferases; HDACs, histone deacetylases; NAD, Nicotinamide adenine dinucleotide; NMN, nicotinamide mononucleotide; ACAT, Acetyl-CoA Acetyltransferase.

Based on these premises, this review will focus on the proposed immune-modulatory approaches that have been tested in vitro or in vivo in chronic hepatitis B and C (Figure 2 and Table 1 and Table 2), targeting:

- innate immunity receptors
- co-inhibitory molecules
- metabolic pathways
- cytokine functions
- epigenetic control of DNA transcription

## 2. Stimulation of Innate Immunity Receptors as an Immunomodulatory Strategy to Overcome HBV-Specific T Cell Exhaustion

Among the possible strategies to develop novel therapies for chronic HBV infection, the reconstitution of the anti-viral T cell function by stimulation of innate immunity receptors, such as toll like receptors (TLRs), retinoic acid-inducible gene 1 (RIG-I) and stimulator of interferon genes (STING), represents a potential approach to rescue protective immunity in CHB patients [71] (Figure 2 and Table 1 and Table 2). These agents are designed to stimulate pattern recognition receptors ultimately leading to the production of cytokines with antiviral activity (e.g., IFNs), with the final purpose of activating host immunity in a non-antigen-specific manner. TLRs are located on the cell surface or within the endosome where they sense molecular patterns associated with viruses and bacteria.

Many attempts to stimulate innate immunity through TLR7 activation have recently been described [31]. TLR7 is predominantly expressed on the endosomal membrane of B lymphocytes and plasmacytoid dendritic cells and its stimulation results in the activation of transcription factors NFkB and IRF7.

Recent studies showed a sustained antiviral response in chimpanzees and woodchucks displayed by the TLR7 agonist GS-9620 [32,33]. A further study conducted in humans by Boni et al. demonstrated that TLR7 stimulation can enhance NK and T cell responses in chronic patients with inhibition of HBV replication induced by nucleos(t)ide analogues (NUC) [34]. Although TLR7 stimulation improved the effect of NUC on T cell responses, this was not sufficient to permit the acquisition of an optimal immune reactivity adequate to provide complete control of infection, as detectable after spontaneous control of self-limited acute infections. This lack of therapeutic efficacy in CHB patients could be widely explained by the very low dose of drug used in humans to avoid possible toxic effects. Recently, a new TLR-7 activating agent, named APR002, has been used alone or in combination with Entecavir (ETV) in the woodchuck model of infection. Importantly, this compound displayed a preferential intrahepatic delivery due to the active uptake by the organic-transporting polypeptide 1B1/3 transporters, that are highly expressed on hepatocytes. Thanks to this liver selectivity, APR002 was highly localized in the woodchuck liver minimizing systemic exposure and promoting a sustained viral control in animals with chronic hepatitis [35,36].

TLR8 is another member of the pattern recognition receptor family, which is preferentially expressed in myeloid dendritic cells (DCs), monocytes, macrophages and Tregs. Recently, much interest was directed to GS-9688 (Selgantolimod), an agonist of endosomal TLR8, whose stimulation leads to the production of immunomodulatory (e.g., IL-12, IL-18) and pro-inflammatory cytokines (e.g., IL-1β, TNF-α), ultimately triggering a strong IFN-γ production by liver resident cells. Through these effects, agonist-induced activation of TLR-8 demonstrated antiviral efficacy both in vitro in HBV-infected primary human hepatocytes and in vivo in woodchucks chronically infected with woodchuck hepatitis virus (WHV) [37,38]. Further insights into the mechanism of action of GS-9688 were provided by investigating its effect in vitro on human immune cells of healthy subjects and CHB patients. TLR8 stimulation activated DCs and mononuclear phagocytes to produce IL-12 and TNF-α, increased the frequency of functional HBV-specific CD8 T cells in a proportion of CHB patients, activated NK cells, mucosal-associated invariant T cells (MAITs), and reduced the frequency of Tregs and myeloid-derived suppressor cells (MDSC) [39]. The safety, pharmacokinetics and pharmacodynamics of Selgantolimod was evaluated in CHB patients in a phase 1b study, in which a good tolerability and a transient dose-dependent elevation of serum IL-12 and IL-1RA were shown, suggesting possible effects on immune responses [40].

Interestingly, dual-acting TLR7/8 (R848) and TLR2/7 (CL413) agonists were described as potent inducers of a broad spectrum of antiviral cytokines able to efficiently inhibit HBV production in HBV-infected primary human hepatocytes, thus providing the rationale for a poly-specific TLR agonist therapy, as a novel approach for HBV cure [41].

More recently, the new immunomodulatory AIC649, an inactivated parapoxvirus-based particle able to stimulate innate immunity via the TLR9 pathway, was shown in combination with Entecavir to significantly decrease viral DNA and WHsAg in the woodchuck model of chronic hepatitis. Suppression of viral replication in the liver correlated with the intrahepatic expression of type I and II IFNs [42]. The importance of the TLR9 signaling as a new target for stimulatory approaches was also emphasized by a recent report demonstrating that host conditioning with the CpG oligonucleotide could restore exhausted intrahepatic CXCR5+ CD8 T cells, via upregulation of co-stimulatory molecules CD80/86 and OX40 on myeloid cells with subsequent increase of CD28-mediated signals on T cells [72].

RIG-I is an RNA sensor present in the cytoplasm, that induces IRF3, IRF7 and NF-kB activation leading to the production of type I and III IFNs, ISGs and pro-inflammatory cytokines. SB 9200 is an oral small dinucleotide that activates RIG-I and nucleotide binding oligomerization domain containing protein 2 (NOD2), resulting in the stimulation of the IFN signaling pathway. Pretreatment with SB 9200 followed by Entecavir administration demonstrated a marked antiviral efficacy and increased the expression of type I IFNs and ISGs in blood and liver in woodchucks chronically infected with WHV [43]. SB 9200 showed antiviral efficacy also against several HCV genotypes and against HCV derived from chronically infected patients who failed to respond to current antiviral treatments [44]. Remarkably, a recent study showed the efficacy of a small IRF3 agonist, called F7, and of a RIG-I agonist derived from a PAMP motif within the HBV genome comprising 50ppp and the poly-U/UC region of viral RNA in suppressing cccDNA in HBV-infected hepatocytes [73]. However, RIG-I stimulation does not represent at present a close therapeutic perspective for chronic hepatitis B since, despite some promising antiviral effects shown in animal models, a phase IIb trial with a RIG-I agonist has been discontinued due to unexpected serious adverse events.

## 3. Boosting Adaptive Immune Response by Blocking Co-Inhibitory Pathways

The role of co-inhibitory molecules in HBV-specific CD8 T cell exhaustion has extensively been described by many in vitro studies on both peripheral and intrahepatic virus-specific CD8 T cells (Figure 2, Table 1 and Table 2). Indeed, in chronic HBV infection, circulating virus-specific CD8 T cells are characterized by the up-regulation of several inhibitory receptors such as PD-1, 2B4, CTLA-4, TIM-3 and LAG-3 with maximal expression of PD-1 in liver-infiltrating HBV-specific T lymphocytes [45,46,53,54,55,56] (Figure 1).

As previously reported in other chronic viral infections, such as LCMV and HCV [4,19,20,25,26,74,75], also exhausted HBV-specific CD8 T cells are a heterogeneous population composed of different subsets with distinct levels of exhaustion according to different HBV antigen specificity that coexist together [21,22,24,27,76]. Due to this heterogeneity, virus-specific CD8 T cells have been reported in other models of T cell exhaustion to display variable susceptibility to immune restoration treatments [19,20,77,78,79,80]. Therefore, identifying those patients who are more likely to benefit from immune-therapy on the basis of their degree of dysfunction may represent a useful strategy to select optimal candidates for functional T cell restoration treatments.

PD-1 is the most widely studied inhibitory receptor in both tumors and chronic viral infections and at present, in the field of chronic HBV infection, strategies aimed at blocking the PD-1/PD-L1 pathway represent the most investigated therapeutic option in vitro, in animal models and in chronically infected patients. Agents that block the PD-1/PD-L1 axis have been described to enhance HBV-specific T cell responses in many in vitro studies, showing a higher level of improvement on the intrahepatic than on the peripheral compartment [46,47,53]. However, T cell functional restoration induced by PD-1/PD-L1 blockade, also in association with the modulation of other co-inhibitory or co-stimulatory pathways, such as CTLA-4, CD137, TIM-3, 2B4, was observed only in a limited percentage of CHB patients with a wide heterogeneity of T cell responsiveness [45,54,55,56]. Interestingly, an increased secretion of IFN-γ and IL-21 producing HBV-specific CD4 T cells has been observed upon OX40 stimulation in combination with PD-L1 blockade, suggesting that immunotherapeutic approaches can also improve HBV-specific CD4 T cell responses [57].

A triple combination therapy of ETV treatment, DNA vaccination and in vivo PD-L1 blockade has been reported in woodchucks infected with WHV to boost virus-specific T cell responses leading to loss of WHsAg and decrease of viral replication [48,49]. In a more recent study, WHV-infected woodchucks were treated with a combination of anti-PD-L1 antibody and NUC. This combinatory approach improved control of viremia and antigenemia compared to ETV treatment alone, but with efficacy restricted to a proportion of animals [50]. The first evidence for a potential benefit of PD-1/PD-L1 blockade in the treatment of chronically infected HBV patients comes from the first clinical trial of anti-PD-1 (Nivolumab) in patients with advanced hepatocellular carcinoma, some of them with HBV infection under antiviral therapy [81]. Indeed, the PD-1 inhibitor nivolumab showed a safety profile devoid of severe hepatic toxicity, with no reactivation of HBV among the cohort of CHB patients. Interestingly, 6% of subjects with chronic HBV infection revealed a substantial decline of HBsAg upon nivolumab treatment.

A recent report extensively reviewed PD-1/PD-L1 blockade immunotherapy clinical trials to investigate the safety and efficacy of this new immunomodulatory strategy in the particular setting of HBV-infected HCC patients. The authors reported acceptable toxicity similar to that of non-infected HCC subjects and promising outcomes. However, due to the standard anti-viral therapies routinely administered in these trials, the antiviral efficacy of anti-PD-1/PD-L1 treatment was not evaluated [51].

Recently, in a phase 1 clinical trial a single low dose of Nivolumab was given to virally suppressed HBeAg negative chronic HBV patients with or without the HBV therapeutic vaccine GS-4774. Of note, significant HBsAg declines were observed in a limited proportion of patients, while one subject showed undetectable HBsAg levels associated with an increase of core- and surface-antigen specific T cell responses [52]. No serious adverse effects were reported likely due to the very low dose (ten times lower than the approved dose for tumors) and the short time of anti-PD-1 administration. Despite these encouraging results, however, the major concern related to PD-1/PD-L1 pathway blockade in chronic HBV infection remains the possibility of severe liver inflammation and hepatitis exacerbation [70]. In addition, the evidence that sustained responses upon PD-1 blockade treatment are detectable only in a limited proportion of HCC and chronic hepatitis B patients suggests the need of developing predictive parameters of response to treatment in order to select the patient cohorts that can benefit of check-point inhibition and to design further modulatory approaches to combine with PD-1 blockade to be used in alternative to it. Along with this line of possible strategies, inhibition of acyl-CoA:cholesterol acyltransferase (ACAT) has recently been reported to significantly improve in vitro HBV-specific CD8 T cell responsiveness to PD-1 blockade pointing to the possibility to combine metabolic and immune checkpoint modulation for the functional cure of HBV infection and HBV-related HCC [69].

## 4. Targeting Metabolism to Restore T Cell Exhaustion

### 4.1. Early Chronic Infections

TCR stimulation elicits multiple intracellular pathways required for metabolic reprogramming and effector function engagement. During T cell activation, glycolysis and mitochondrial dynamics are central for fine-tuning metabolic switching to meet the energetic requirements of T cells in response to nutrients. The metabolic plasticity is a key feature of T cell activation and it is fundamental for the responsiveness to changes in the tissue microenvironment [82,83].

During acute infection, CD8 T cells shift from mitochondrial oxidative phosphorylation distinctive of the quiescent state of naïve T cells, to a glycolysis-driven energetic metabolism necessary for activated effector T cells. This remarkable metabolic reprogramming, known as Warburg effect or aerobic glycolysis, is mandatory to meet the initial proliferative burst and it is due to phosphoinositide 3-kinase (PI3K) and mTOR signaling pathways triggered by TCR and CD28 co-stimulation [82,84,85]. After completion of the effector phase, developing memory T cells need to shift back to oxidative metabolism guided by mitochondrial fatty acid degradation. On the other hand, during chronic viral infection, long-term antigen exposure drives a specific T cell differentiation program [82,83,86,87].

Transcriptional and functional profiles of developing exhausted T cells display metabolic derangements, including suppressed glycolytic functions, reduced cellular respiration associated with diminished glucose uptake, with a final marked decline in ATP generation. This early scenario is evolutionarily maintained in early exhausted T cells in both murine (i.e., LCMV) and human (i.e., chronically-evolving acute HCV infection) virus infection, but the transcriptional dysregulated signature is less conserved in established exhausted T cells across different species and different models of infection, including HCV [64,88,89], where functional enriched modules have been associated to cell cycle replication, RNA processing, intracellular signaling and histone methylation processes [64].

Therefore, when severe exhaustion is not established yet, CD8 T cells appear to be unable to meet the energy requirement for effector function maturation. Indeed, they show reduced glucose utilization associated with high anabolic signal levels, as indicated by the strong upregulation of genes involved in class I PI3K–AKT-mTOR signaling linked to CD28 upregulation [88], which may cause TCR-signaling defects resulting in a high expression of the PD-1 co-inhibitory receptor. This derangement skews precursors of exhausted T cells to an altered metabolic profile susceptible to mitochondrial depolarization and high reactive oxygen species (ROS) production that contribute to the activation of stress sensor signaling pathways associated with the inhibition of TCR signalosome and of telomerase activity [64].

Several deregulated signaling pathways and molecules are involved in these early metabolic defects, including the peroxisome proliferator-activated receptor γ (PPARγ) coactivator 1α (PGC-1α) [88], which is a key transcriptional regulator of mitochondrial biogenesis-related genes [4,90], as well as DNA-repair and metabolic effectors, such as p53/ATM and AMPK/p38, reported in HCV infection [64]. Indeed, PGC-1α expression is significantly reduced in early exhausted virus-specific CD8 T cells, which express high levels of PD-1 and display depolarized mitochondria with a distinctive morphology resulting from mitochondria accumulation and fusion which lead to ROS overproduction [88]. In vivo, PGC-1α overexpression corrected the dysregulated mitochondrial phenotype and significantly increased polyfunctionality of CD8 T cells, by improving the metabolic cell fitness in chronic LCMV infection [88].

During early acute HCV infection two different studies described transcriptional derangements related to DNA repair and histone methylation processes in early exhausted T cells [64,89]. Due to the high antigenemia, a continuous TCR stimulation activates proliferative signals which require a sustained DNA repair response to fix the resulting DNA damages. In an effort to elucidate the mechanisms behind the poor metabolic fitness described in early exhausted CD8 T cells, different regulatory pathways responsible for metabolic alterations have been described allowing to define possible targeting strategies for their correction. For instance, a specific inhibition of downstream hyper-expressed effectors of the activation signaling cascade in HCV-specific CD8 T cells elicits a functional metabolic and antiviral T cell reconstitution in the majority of tested patients [64]. This CD8 T cell restoration effect can be achieved in chronically evolving acute HCV infection by blocking p53, a master regulator of cell-cycle arrest and metabolic reprogramming, activated in response to replicative stress, which can ultimately cause cellular apoptosis in case of irreparable insult [64,91,92]. Similar effects have been reported by inhibiting the upstream and downstream effectors of p53 [64,93], including ataxia telangiectasia mutated kinase (ATM), the major activator of the DNA damage response [94], activated during energetic stress, and p38 MAPK, a final effector molecule which can inhibit TCR-signaling and telomerase functionality [64,95,96] (Figure 2 and Table 1 and Table 2).

Signaling deregulation in the acute phase of chronically evolving HCV infection is possibly guided by ROS overproduction resulting from an oxidative metabolic reprogramming modulated by oxidative-instructive signals on mitochondrial activity that have been reported by both PD-1 and p53 [91,97]. Indeed, lymphocyte treatment with anti-oxidant molecules, such as resveratrol and N-acetyl-L-cysteine (NAC), can reduce intracellular ROS levels with a resulting activity decline of the upstream replicative-stress sensor ATM kinase [64]. Thus, the energy unbalance associated with low glycolytic levels and mitochondrial derangement, can increase DNA damage through ROS over-production, cell-cycle arrest, ultimately resulting either in apoptosis of early exhausted lymphocytes or in the survival of terminally exhausted poorly functional CD8 T cells.

Mitochondrial ROS-induced DNA damage accumulation results in the persistent activation of DNA repair mechanisms, including PARP enzymes [98], as suggested by the up-regulation of PARP-2 and PARP-1 transcripts in chronically evolving as compared with self-limiting HCV infections [64]. PARPs utilize NAD molecules as donors of poly-(ADP)-ribose monomers to catalyse the formation of poly-(ADP)-ribose chains that mediate early recruitment of DNA repair factors in correspondence of the DNA breaks [99]. Persistent PARP activation, induced by persistent DNA damage, can lead to a profound consumption of the cellular NAD pool, which can affect different metabolic and signaling pathways (see below).

### 4.2. Established Chronic Infections

Comparison of early- and late-stages of chronic HCV and HBV infections by transcriptomic analyses of virus-specific CD8 cells [26,64,65,89] revealed completely divergent profiles of early and late exhausted CD8 T cells: upregulated genes were highly enriched in early exhausted cells derived from the acute phase of HCV infection, while a massive gene downregulation was prevalent in late exhausted cells of the established chronic phase of both infections [64,65].

Indeed, in long term chronic infections, an extensive transcriptional reprogramming with global repression in gene expression has been described in both hepatitis B and C (Figure 1), as a likely survival strategy which can allow exhausted lymphocytes to endure continuous antigenic stimulation that leads to the progressive loss of effector functions.

In both chronic infections the most significantly downregulated cellular pathways and functions are relative to mitochondria, proteostasis, DNA repair and transcription [64,65] (Figure 1). Interestingly, these altered processes are mechanistically interconnected and, at least in part, attributable to NAD shortage (personal communication). Indeed, in addition to hyper-stimulation of PARPs activity by accumulated DNA damage, that can take place in the acute phase of infection, CD38, a major NAD+ hydrolyzing enzyme, is expected to play a central role in NAD consumption and depletion [100], since it is overexpressed in both HBV- and HCV-specific CD8 T cells in the acute and chronic stages of infection [64,65] (Figure 1). NAD depletion can cause reduced activity of sirtuins, which are NAD-dependent deacetylases with multiple regulatory roles [101]. In addition, NAD shortage can influence epigenetic control of DNA transcription both by limiting sirtuins’ activity [102,103,104] and by metabolic modifications. Indeed, lowered NAD/NADH ratio can induce a preferential conversion of pyruvate to lactate and decreased formation of acetyl-CoA available for histone acetylation [105], leading to chromatin condensation and gene silencing, in line with the global gene expression downregulation observed in chronic hepatitis B and C [64,65] (Figure 1). Along this line, CD38 overexpression causing NAD shortage and sirtuin 1 activity reduction has been demonstrated to cause the increased activity of the methyltransferase EZH2 in dysfunctional T cells from patients with systemic lupus erythematosus, leading to enhanced histone methylation [104]. This mechanism is likely to be active also in virus-specific CD8 T cells during chronic hepatitis C, where an enhanced histone methylation by EZH2 has been observed [64].

Reduced sirtuin activity also contributes to mitochondrial dysfunction, through increased mitochondrial ROS and impaired mitophagy and may affect directly several DNA repair pathways, generating a vicious cycle leading to persistent DNA damage and NAD consumption [106].

In addition, generation of the sirtuin-inhibitor nicotinamide as a product of NAD hydrolysis by CD38 [107], can lead to loss of sirtuin function, further exacerbating DNA damage and mitochondrial dysfunction [106,108].

Given the key role suggested by these findings for NAD depletion in CD8 T cell exhaustion pathogenesis, replenishment of the depleted NAD pool may represent a possible strategy to be pursued in the future to restore the anti-viral function of exhausted CD8 T cells.

In the persistently infected liver different inhibitory signals originate from the peculiar intrahepatic environment, contributing to further repress exhausted T cells by influencing T cell metabolism and function.

Amino acid starvation is one of the mechanisms described to inhibit T cell proliferation, since damaged hepatocytes and other infiltrating immune cells, can release enzymes able to degrade amino acids, like arginine or tryptophan. Indeed, hepatocytes constitutively express tryptophan-2,3-deoxygenase (TDO), that catalyzes the first limiting step of tryptophan degradation oriented to the biosynthesis of NAD, producing toxic intermediates collectively known as kynurenines [109] (Figure 1). Hepatocyte damage can lead to tryptophan depletion together with the massive production of kynurenines, that have been demonstrated to inhibit T and NK cell proliferation. T cell expansion at the intrahepatic level can be critically affected also by shortage of another essential amino acid, L-arginine, due to an excess of arginase derived from damaged hepatocytes [109], leading to T cell arrest in the G0/G1 phase and CD3ζ downregulation (Figure 1). In chronic HBV infection a subset of arginase-expressing myeloid-derived suppressor cells (MDSC) has been found particularly expanded in the liver of patients with high viral load and low liver inflammation [68]. In these patients, arginine depletion leads to compensative up-regulation of amino acid transporters on T cells, to enhance essential amino acids up-take. The relevance of these mechanisms is confirmed by the evidence that arginine replenishment, as well as the addition of an arginase inhibitor, can partially restore the T cell function [68].

Moreover, the high intrahepatic cholesterol concentration is a factor likely able to influence T cell metabolism and function, as in immune cells lipid accumulation has been reported to affect the cellular function.

In line with this, in chronic hepatitis B inhibition of cholesterol esterification by the acyl-CoA:cholesterol acyltransferase (ACAT), that catalyzes the esterification of excess intracellular cholesterol for storage in lipid droplets, enhanced TCR signaling upon CD8 T cell stimulation, by reducing lipid droplet formation and diverting cholesterol to the cell membrane [69] (Figure 2, Table 1 and Table 2). This strategy has been shown to be active both to enhance HBV- and HCC- specific T cell proliferation and effector functions, by optimizing cells bioenergetics, and to exert an antiviral effect by reducing HBV virions and viral particles formation in vitro [69].

The intrahepatic environment can also influence autophagy up-regulation in tissue-resident T cells, which is needed to counterbalance mitochondria depolarization and thus maintain mitochondrial fitness and T cell functionality in such a condition of high oxidative stress, as autophagy inhibition was shown to induce depolarized mitochondria accumulation [58]. Indeed, HBV-specific CD8 T cells in chronic hepatitis B have been shown by both transcriptional and functional validation analysis to display defective autophagy and dysfunctional mitochondria with low membrane potential and high ROS production [65] (Figure 1). Interestingly, IL-15 and the natural polyphenolic compounds resveratrol and oleuropein not only increased the cellular proteostasis, but could also restore the mitochondrial function, thus leading to an improved antiviral T cell function in CHB patients [58,66] (Figure 2, Table 1 and Table 2).

The presence of depolarized mitochondria is indeed a typical feature of exhausted T cells, detected both in chronic hepatitis B and C, which involves a lower respiratory capacity for energy generation [64,65]. As a consequence, in chronic HBV infection HBV-specific CD8 T cells rely upon a compensatory glycolytic function, with up-regulation of the glucose transporter GLUT-1, in an attempt to counterbalance dysfunctional mitochondria [59,65] (Figure 1). Administration of the cytokine IL-12 could reverse mitochondria depolarization and glycolysis dependence, finally improving the antiviral T cell function [59] (Figure 2, Table 1 and Table 2). Also mitochondria-targeted (mt-)antioxidant compounds led to an improved mitochondrial fitness and T cell cytokine production in T cells isolated from patients with chronic hepatitis B by neutralizing excess ROS production in vitro [65,66]. The mt-antioxidant MitoQ has been tested also in vivo in patients with chronic HCV infection not responding to PEG-interferon plus ribavirin treatment, in a phase II study. It showed lack of toxicity and induced a decrease in ALT levels, showing liver protection activity, although not reducing the viral burden [67] (Figure 2, Table 1 and Table 2).

## 5. Cytokine Fueling for T Cell Restoration

An important aspect of cytokine therapeutic usage relies on targeting both intracellular signaling and metabolic dysfunctions detected in T cell exhaustion, with the possibility of metabolic reprogramming (Figure 2, Table 1 and Table 2).

IL-2 secretion from CD8 T cells occurs early after TCR engagement. IL-2 boosts AKT1 and mTORC1 activation through IL-2Rα (CD25) triggering, thereby conveying signals to promote glucose and amino acid uptake as well as protein and lipid biosynthesis, required for proper T cell effector functions [110,111,112,113].

In vivo treatment with recombinant IL-2 was recently reported to induce better T cell effector differentiation of HBV-specific CD8+ T cells. Studies were conducted in Iannacone’s lab through the generation of unique mice models which express HBV antigens either selectively in hepatocytes (MUP-core mice), causing defective antigen presentation and lack of differentiation in functional effector CD8 T cells, or only in Kupffer cells and hepatic dendritic cells (HDCs) with efficient antigen presentation leading to proper and functional differentiation of effector CD8 T cells [61]. Through IL-2 supply, defective virus-specific CD8+ T cells restored the ability to proliferate and differentiate in IFN-γ producing cells, whilst anti-PD-L1 alone administration was not effective [61]. Moreover, IL-2 treatment was able to restore the transcriptional program of the dysfunctional virus-specific CD8+ T cells from MUP-core mice livers, as revealed by RNA-seq experiments [61]. The therapeutic efficacy was also confirmed by IL-2 treatment in HBV replication-competent transgenic mice, providing evidence of cytotoxic and IFN-γ secreting core-specific CD8+ T cell differentiation in the liver. Results in this mouse pre-clinical model were then assessed in different HBV infected patient categories. Only HBV-specific T cells derived from immune-tolerant patients, which more closely resemble T cells that have been primed by hepatocytes, were in fact rescued by IL-2 addition in vitro, acquiring proper IFN-γ production levels observed in immune-active patients, which were not boosted by IL-2 addition [61]. These conclusions indicate that the preclinical mouse model developed in Iannacone’s lab nicely reflects the pathogenetic mechanisms involved in neonatal infection and point to the relevance of conducting specific trials in selected cohorts of chronic HBV patients identified on the basis of the degree of exhaustion. In vivo use of IL-2 is, however, not recommended for systemic toxicity [112]. Therefore, a modified version of IL-2 (named IL-2c), previously shown to have reduced toxic systemic effects and able to selectively target CD8 T cells [112], has been employed as a new immunotherapeutic tool in the context of chronic HBV infection [61].

Another therapeutic option is provided by the pro-inflammatory cytokine IL-12 that has been shown to be able to improve the function of HBV-specific CD8 T cells with an augmented effect when tested in combination with PD-1 blockade. IL-12 alone was effective in restoring both polyfunctional secretion of multiple cytokines and cytotoxic activity in HBV-specific CD8+ T cells due to the increased expression of the cytokine-regulatory transcription factor Tbet, along with decline of PD-1 and of the pro-apoptotic Bim expression, with the consequent reduction in Bim-mediated apoptosis [60]. In addition, IL-12 could efficiently enhance mitochondrial fitness with better control of mitochondrial membrane potential and reduction in the percentage of depolarized mitochondria and with restoration of IFN-γ production in HBV-specific CD8 T cells [59].

In vivo systemic IL-12 administration, such as in cancer trials, has poorly been tolerated but the use of TLR-8 agonists (e.g., GS-9688), which stimulate the production of pro-inflammatory cytokines, including IL-12, is an attractive alternative in combination with a therapeutic DNA vaccine for CHB patient treatment [114,115,116].

The magnitude of T cell effector responses is augmented when CD8 cells are activated by the combination of IL-12 and IL-2 by virtue of a functional synergy on STAT1 and STAT3 phosphorylation observed in activated T cells but this combination has never been tested in CHB patients [117].

A promising molecular target for exhausted T cells in CHB patients is represented by IL-15, which has been reported in vitro to increase the autophagic activity of tissue resident memory lymphocytes [58]. Given the deficiency of lysosome-mediated autophagy observed in HBV-specific CD8 T cells [66], IL-15 use in chronic HBV patients deserves to be tested for its capacity to restore the anti-viral CD8 T cell function.

In CHB patients an interesting immune approach for metabolic T cell reconstitution consists of PGE2 signaling blockade. PGE2 has been shown to contribute to CD8 T cell exhaustion development during viral infections both in mice and in humans [63]. Indeed, blockade of PGE2 signaling in the AAV-HBV1.2 mice model reduced TIM3 expression on total CD8 T cells and induced restoration of the CD8+ T cell function and control of HBV infection [63]. However, the exact mechanisms for PGE2 regulation on CD8 T cells is still debated in chronic HBV infection and needs to be elucidated.

In the setting of chronic HCV infection IL-7 has been tested in vitro. It was shown to be able to correct the downregulation of the TNF receptor-associated factor 1 (TRAF1), which correlates with the exhaustion phenotype in HCV-specific CD8 T cells. Moreover, IL-7 could improve HCV-specific CD8 T cell proliferation capacity when added to triggering of the positive costimulatory receptor 41BB, and even more when also PD-L1 blocking was associated, particularly in patients with slowly progressing fibrosis [62].

## 6. T Cell Epigenetic Targets for Therapies during Chronic Infections

A great research effort has been devoted in the last years to understand the relationship between metabolism and T cell differentiation programs [118,119,120]. The comprehension of how metabolic changes can impact on the epigenetic control of gene expression is key to understanding the T cell differentiation process during persistent viral infections. Epigenetic mechanisms are crucial for T cell differentiation by acting directly on gene expression profiles, thereby representing a multilayered regulation between signaling events, metabolic reprogramming and differentiation gene programs in T cells. TCR and IL-2 signalings coordinate effector T cell activation through the induction of the Myc oncogene, a transcription factor which controls expression of genes encoding transporters and enzymes involved in the glycolysis and glutaminolysis pathways, which regulate ribosome biogenesis by amplifying gene expression [119,121,122,123,124]. Moreover, the TCR-inducible transcription factors, SREBPs (sterol binding proteins), can activate expression of components of the lipid biosynthesis pathway that in the early stages of T cell induction is fundamental for generating new cellular membranes which are necessary for rapid T cell division [125,126]. Other key transcription factors are members of the hypoxia-inducible factors (HIFs) which are directly regulated by IL-2-dependent events, with a further positive feedback for genes that encode glycolysis and glutaminolysis components [127,128].

In this perspective, transcription factors which represent signaling mediators, provide regulating decisions fundamental for metabolic gene reprogramming, which depends also on the nutrient supply present in the microenvironment [129]. All these aspects have strong influence on the proper cellular differentiation program during viral infections.

The correct metabolic reprogramming during T cell differentiation is obviously central for the resultant availability of metabolites which could serve as substrates, donors, cofactors and antagonists for epigenetic-modifying complexes and consequently for the overall epigenetic status.

For instance, glycolysis is the natural primary pathway used to generate the acetyl-CoA cofactor during T cell differentiation [130]. The enzyme LDHA (lactate dehydrogenase A) transforms pyruvate in lactate to avoid oxidation of glucose in the mitochondria, leading to accumulation of citrate in the cytosol, which is then converted into acetyl-CoA by the enzyme ATP-citrate lyase, ultimately ending in the enhancement of histone acetylation modifications, such as histone H3 lysine 9 (H3K9Ac) and histone H3 lysine 27 (H3K27Ac) in the IFN-γ locus, to boost T cell effector gene expression [130,131]. This provides a link between glycolysis, acetyl-CoA and histone acetylation in the regulation of T cell differentiation. Thus, understanding the fine tuning of enzymes that modify histones or DNA such as “writers” which add chemical groups (e.g., histone acetyltransferases—HATs, histone methyltransferases—HMTs, and DNA methyltransferases—DNMTs) and “erasers” which remove them (histone deacetylases—HDACs, and Tet enzymes) will be necessary for the design of correct and effective strategies to reconstitute T cell function and fate by chromatin modulation and epigenetic inhibitor strategies for immune-related diseases.

Expanding studies based on assays with high-throughput sequencing for transposase-accessible chromatin (ATAC-seq) and for chromatin immunoprecipitation (ChIP-seq) have been carried out to characterize histone modifications and chromatin accessibility in immune cells at different stages of development [74,75,132,133,134,135]. By genomic profiling, the transcriptional and epigenetic landscape of exhausted T cells has been elucidated and shown to be endowed with a distinct transcriptional program compared to effector and memory T cells. Exhausted CD8 T cells diverge from other CD8 T cells by ~6000 differentially accessible chromatin regions which are linked to differentially expressed genes regulated by distinctive molecular circuits of transcription factors [75,134,136]. Also in HBV infection derangement of transcriptional factors has been identified with the discovery of up-regulation of negative-regulator of transcription and closed chromatin configuration in HBV-specific CD8 T cells from chronic patients [65]. Integrated network analyses of exhausted T cells showed an altered transcriptional connectivity of pivotal transcription factors, such as Tcf-1 (T-box factor) and Eomes, which are expressed both in functional effector/memory T cells and in exhausted T cells with different transcriptional circuits, leading to different gene expression patterns guided by context-specific activities [15,64,89,137,138]. This scenario strengthens the concept that the epigenetic landscape of exhausted T cells is a stable condition, early induced during development of exhaustion, which regulates responsiveness to checkpoint blockade treatment [75,134,139,140]. As already discussed, checkpoint blockade (e.g., on PD-1) can induce functional reinvigoration of CD8 T cells which is associated however with limited changes in the epigenetic state, explaining why cells tend to revert back to the functional and transcriptional features of the prior exhausted condition [75].

Importantly, highly condensed regions of heterochromatin contain few active genes and are characterized by high levels of histone modifications, such as tri-methylation of histone H3 lysine 9 (H3K9me3), tri-methylation of histone H3 lysine 27 (H3K27me3) and low levels of histone acetylation [141].

As previously described, a distinctive transcriptional pattern has been shown in dysfunctional CD8 T cells with a prevalent downregulation of gene expression also in human chronic hepatitis infections [64,65,89]. Conversely, by investigating epigenetic-modifying complexes, early during chronically-evolving acute infections a dysregulated over-expression of histone methyltransferases (e.g., G9a and EZH2 HMTs) has been observed [64]. One of the best-known methyltransferase complexes active in T cell differentiation is the polycomb repressive complex (PRC2) [142], whose catalytic subunits are EZH2 and EZH1, which are responsible for H3K27me3 histone modification and thus for a non-permissive chromatin signature [143].

In response to a euchromatic DNA double-strand break (DSB), such as the DNA breaks occurring as a result of the high proliferation rate of CD8 T cells in early acute viral infections, an ATM-mediated signal is sent to repress transcription in flanking chromatin regions [144] which is catalyzed by polycomb complex proteins [145,146]. Subunits present in PRC complexes are then recruited to the sites of DNA damage to help DNA repair mechanisms, mediating DSB-induced transcriptional silencing [147,148,149,150]. This scenario is similar to that observed in the early phases of persisting HCV infection, where increased expression and activity of ATM, p53 and HMTs have been observed [64].

Moreover, several studies described key roles for PRC2 and G9a HMT in CD4 and CD8 T cell differentiation for effector and memory commitment [132,151,152,153,154].

G9a (EHMT2) is a lysine methyltransferase whose primary function is to di-methylate histone H3 lysine 9 (H3K9me2) by acting primarily through the recruitment of H3K9me2-binding proteins that prevent transcriptional activation [155]. In addition, G9a directly interacts with Blimp-1, a zinc finger protein that has been shown to repress key aspects of normal memory CD8+ T cell differentiation and to promote high expression of inhibitory receptors during T cell exhaustion in chronic viral infection [14]. Moreover, G9a can directly repress type I cytokine gene expression, such as the IFN-γ locus during Th2 differentiation by regulating both H3K9me2 and H3K27me3 [156].

An intriguing study recently showed that CD8+CD38^high^ T cells displayed increased expression of EZH2 compared with CD8+CD38^low^, by pinpointing the underlying mechanism responsible for a decreased cytotoxicity of the cells primarily due to low NAD levels which alters chromatin organization [104].

A functional inhibition of HMTs by treatment with specific EZH2 and G9a inhibitory compounds, led to a T cell functional reconstitution both as anti-viral cytokine production and as proliferative capacity, with a partial metabolic restoration [64] (Figure 2, Table 1 and Table 2). Although CD8 T cell functional restoration has been observed in both acute and chronic stages of HCV infection, the highest stimulatory effect on HCV-specific CD8 cells was observed when chronicity was already established [64]. Moreover, in tumor-bearing mice a synergistic effect has been seen between several epigenetic inhibitors (e.g., blockade of EZH2) and check-point blockade treatments (such as with anti-CTLA-4 and anti-PD-1 treatment) with respect to their immune-modulatory actions [157,158].

The picture of T cell exhaustion that emerges from HCV and HBV infections, thus, highlights a profound and wide-ranging cellular perturbation centered on metabolic and signaling deregulation that results in a global scenario of chromatin remodeling leading to a complete repression in gene expression characteristics of the established T cell exhaustion (Figure 1). Therefore, epigenetics drugs represent potential and promising candidate for immune modulatory therapies for chronic viral infections which may have the capacity to restore several T cell functions together.

## 7. Future Perspectives

Stress responses and deregulated metabolic pathways can severely impair T cell immunity during chronic viral hepatitis. The comprehension of these alterations in metabolism, signaling and epigenetics will certainly open new horizons for the definition of novel immunometabolic targets to restore proper T cell differentiation pathways. Particular attention should be devoted to the possible modulation of epigenetic mechanisms and to circumvent the potent tolerogenic effect exerted by the liver microenvironment, where elevated antigen loads can persistently exert high pressure on T cell responses, skewing them towards the activation of inhibitory intracellular circuits.

Also virus-specific T cell heterogeneity should be better decrypted, perhaps making use of the artificial intelligence to discover novel molecular targets for immune modulatory therapies and novel predictive parameters to identify patients more likely responsive to modulatory compounds.

## Figures and Tables

**Table 1:** Novel immuno-therapeutic approaches for HBV and HCV chronic infections.

Immune-Modulatory Interventions	Class of Agents	HBV	HCV
Stimulation of innate immunity receptors	GS-9620	[31,32,33,34]	-
	APR002	[35,36]	-
	GS-9688/selgantolimod	[37,38,39,40]	-
	R848	[41]	-
	CL413	[41]	-
	AIC649	[42]	-
	SB 9200	[43]	[44]
Co-inhibitory pathways blocking	PD-1	[45,46,47,48,49,50,51,52,53]	[45]
	CTLA-4	[54]	-
	TIM-3	[55]	-
	2B4	[56]	-
Co-stimulatory signaling activation	CD137	[45]	[45]
	OX40	[45,57]	[45]
Cytokine fueling	IL-15	[58]	-
	IL-12	[59,60]	-
	IL-2	[61]	-
	IL-7	-	[62]
	PGE2 inhibition	[63]	-
Metabolic modulation	p53, p38, AMPK, ATM inhibitory compounds	-	[64]
	N-acetyl-L-cysteine (NAC)	-	[64]
	MitoQ/MitoTempo	[65,66]	[67]
	Polyphenols Resveratrol and Oleuropein	[65,66]	-
	Arginine replenishment	[68]	-
	Acyl-CoA:cholesterol acyltransferase (ACAT) inhibitor	[69]	-
Epigenetic intervention	Histone methyltransferses inhibitors	-	[64]

**Table 2:** Combination Therapies based on immune-modulation for T cell restoration.

Class of Agents	In Vitro/ In Vivo Use	Mechanism of Action	Target Cells	Disease	References
GS-9620	In vivo	Induction of TLR7 activation	-	HBV	[31,32,33,34]
APR002	In vivo	Induction of TLR7 activation	-	HBV	[35,36]
GS-9688/selgantolimod	In vitro/ In vivo	Agonist of endosomal TLR8	Liver resident cells (activated DCs, mononuclear phagocytes and immune cells)	HBV	[37,38,39,40]
R848	In vitro	Dual-acting TLR7/8 agonist	Hepatocytes	HBV	[41]
CL413	In vitro	Dual-acting TLR2/7 agonist	Hepatocytes	HBV	[41]
AIC649	In vivo	TLR9 pathway activator	-	HBV	[42]
SB 9200	In vivo	RIG-I agonist	-	HBV, HCV	[43,44]
Checkpoint blockade	In vitro	PD-1, CTLA-4, TIM-3 and 2B4	T cells	HBV, HCV	[45,46,47,53,54,55,56]
CD137 and OX40	In vitro	Co-stimulatory CD137 or OX40 signaling activation	T cells	HBV, HCV	[45,57]
Nivolumab	In vivo	Anti-PD-1 for PD-1/PD-L1 blockade	T cells	HBV	[48,49,50,51,52,70]
p53, p38, AMPK, ATM inhibitory compounds	In vitro	Stress-sensor signaling kinase blockade	T cells	HCV	[64]
N-acetyl-L-cysteine (NAC)	In vitro	Anti-oxidant compounds	T cells	HCV	[64]
MitoQ/MitoTempo	In vitro/ In vivo	Mitochondrial anti-oxidant treatment	T cells	HBV, HCV	[65,66,67]
Polyphenols Resveratrol and Oleuropein	In vitro	Mitochondrial function and intracellular proteostasis restoration	T cells	HBV	[65,66]
Histone methyltransferses inhibitors	In vitro	EZH2 and G9a blockade	T cells	HCV	[64]
Arginine	In vitro	Arginine replenishment	T cells	HBV	[68]
acyl-CoA:cholesterol acyltransferase (ACAT) inhibitor	In vitro	Inhibition of cholesterol esterification	T cells	HBV	[69]
IL-15	In vitro	Cellular proteostasis and mitochondrial function restoration	T cells	HBV	[58]
IL-12	In vitro	Reverse mitochondria depolarization and glycolysis dependence	T cells	HBV	[59,60]
IL-2	In vitro/ In vivo	T cell proliferate and differentiation restoration	T cells	HBV	[61]
IL-7	In vitro	Exhaustion characterization and TRAF1 restoration	T cells	HCV	[62]
PGE2 inhibition	In vitro	PGE2 inhibitory signaling blockade	T cells	HBV	[63]
